# A Fifth Metatarsal Fracture or a Rare Anatomical Variant?: A Report of Two Cases of Symptomatic Os Vesalianum Pedis and a Review of the Literature

**DOI:** 10.15388/Amed.2025.32.2.17

**Published:** 2025-12-30

**Authors:** Kristina Petrova, Svetlomir Rangelov, Lyubomir Gaydarski, Boycho Landzhov, Georgi P. Georgiev

**Affiliations:** 1 Department of Anatomy, Histology and Embryology, Medical University, Sofia, Bulgaria; 2 Department of Orthopedics and Traumatology, University Hospital Queen Giovanna – ISUL; Medical University, Sofia, Bulgaria; 3 Department of Anatomy, Histology and Embryology, Medical University, Sofia, Bulgaria; 4 Department of Anatomy, Histology and Embryology, Medical University, Sofia, Bulgaria; 5 Department of Orthopedics and Traumatology, University Hospital Queen Giovanna – ISUL; Medical University, Sofia, Bulgaria

**Keywords:** accessory bones, Os vesalianum pedis (OVP), lateral foot pain, differential diagnosis, conservative treatment, papildomi kaulai, *Os vesalianum pedis* (OVP), šoninis pėdos skausmas, diferencinė diagnozė, konservatyvus gydymas

## Abstract

**Background:**

Accessory bones of the foot are common anatomical variants, with *Os Vesalianum Pedis* (OVP) representing a rare example located near the base of the fifth metatarsal within the peroneus brevis tendon. Although typically asymptomatic, OVP can become painful following trauma and may be misdiagnosed as a fracture, leading to inappropriate management.

**Case presentation:**

We present two cases of symptomatic OVP in patients who reported lateral foot pain after acute ankle inversion injuries. Following radiological evaluation at a regional polyclinic, both patients were initially diagnosed with fractures of the fifth metatarsal base and referred to a traumatologist. However, further physical examination and detailed review of previous radiographs, revealing well-corticated, smoothly contoured ossicles, led to the correct diagnosis of OVP. Both patients were treated conservatively with rest, ice, elevation, physiotherapy, and NSAIDs, achieving complete functional recovery within three weeks.

**Conclusions:**

These cases highlight the importance of recognizing OVP as a potential cause of lateral foot pain after trauma. Accurate diagnosis based on imaging characteristics can prevent mismanagement. Conservative treatment remains highly effective, and awareness of OVP is essential for appropriate clinical decision-making.

## Abstract (it)

**Įvadas:**

Pėdos papildomi kaulai yra dažni anatominiai variantai, o *Os vesalianum pedis* (OVP) yra reta, papildoma pėdos kaulų dalis, dažniausiai esanti netoli penktojo pėdos kaulo pagrindo, *peroneus brevis* sausgyslėje. Nors paprastai OVP yra besimptomis, po traumos jis gali tapti skausmingas ir būti klaidingai diagnozuotas kaip lūžis, dėl ko gali būti netinkamai gydoma.

**Atvejo aprašymas:**

Pateikiame du simptominio OVP atvejus, kai pacientai skundėsi šoniniu pėdos skausmu po ūmių kulkšnies išnirimo traumų. Atlikus radiologinį tyrimą regioninėje poliklinikoje abiem pacientams iš pradžių buvo diagnozuotas penktojo pėdos kaulo pagrindo lūžis ir jie buvo pasiųsti pas traumatologą. Tačiau po tolesnio fizinio tyrimo ir išsamaus ankstesnių rentgenogramų peržiūrėjimo, atskleidusio gerai išsivysčiusius, lygius kaulų kontūrus, buvo nustatyta teisinga OVP diagnozė. Abu pacientai gydyti konservatyviai, taikant poilsį, ledą, pakėlimą, fizioterapiją, ir per tris savaites pasiekė visišką funkcinį pasveikimą.

**Išvados:**

Šie atvejai rodo, kaip svarbu pripažinti OVP kaip galimą šoninio pėdos skausmo po traumos priežastį. Tiksli diagnozė, pagrįsta vaizdo diagnostikos charakteristikomis, gali padėti išvengti netinkamo gydymo. Konservatyvus gydymas išlieka labai veiksmingas, o žinios apie OVP yra būtinos tinkamiems klinikiniams sprendimams priimti.

## Introduction

Accessory bones in the foot are relatively common anatomical variations, with a reported incidence of up to 26% [1,2]. One rare example is *Os Vesalianum Pedis* (OVP), an accessory ossicle located proximally to the base of the fifth metatarsal within the tendon of the peroneus brevis muscle [3]. This ossicle results from the failure of fusion between the secondary and primary ossification centers of the fifth metatarsal [3]. The reported prevalence of OVP varies in the literature, ranging from 0.1% to 5.9% [4,5], with Candan et al. estimating a prevalence of 0.3% in their study [2]. OVP is typically asymptomatic and often discovered incidentally [6]. However, it may become symptomatic, causing lateral foot pain in association with trauma, infection, or degenerative changes [6,7]. Accurate diagnosis is essential, as OVP can be mistaken for fractures or other anatomical variants. The differential diagnosis primarily includes avulsion fractures of the fifth metatarsal base, Jones fractures, and other accessory ossicles [8]. Imaging studies, particularly plain radiographs (X-ray), play a central role in identifying and differentiating this condition [2]. This case report describes two patients presenting with symptomatic OVP and provides a brief review of the literature, with particular attention to differential diagnosis.

## Case Presentation

We report two rare cases of symptomatic OVP in a 20-year-old male and a 43-year-old female. Both patients were initially diagnosed with fractures of the fifth metatarsal base at a regional polyclinic following trauma and were subsequently referred to a traumatologist.

The patients presented to the emergency department after acute inversion injuries of the ankle. On clinical examination, both individuals exhibited localized swelling and marked tenderness over the lateral aspect of the foot, corresponding to the insertion of the peroneus brevis tendon. Neither patient had a history of prior symptoms in the affected area. Pain was provoked by inversion and plantarflexion movements, which also resulted in a reduced range of motion. No joint instability or neurovascular deficits were identified.

**Figure 1:**
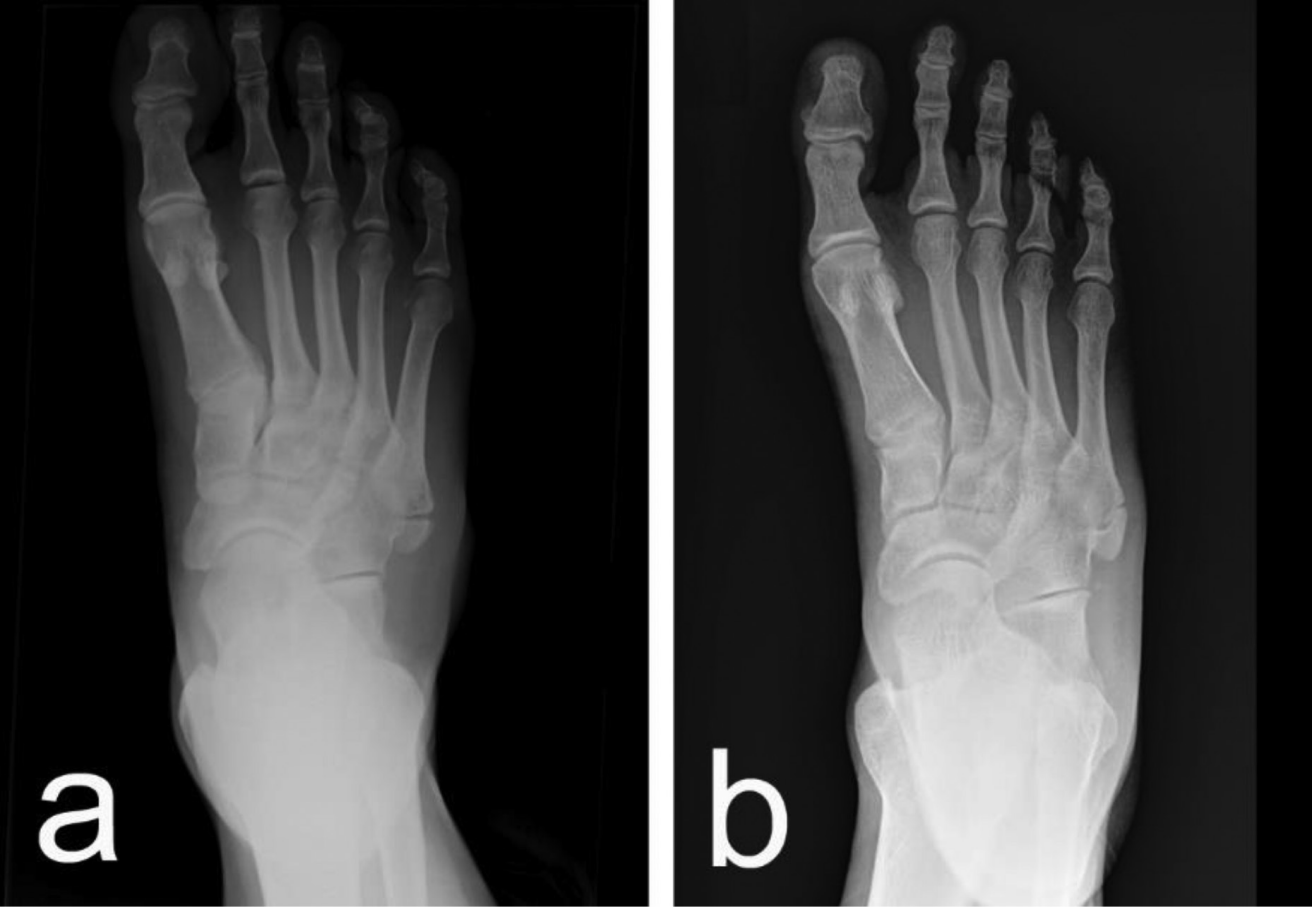
Plain radiographs of the two reported cases of symptomatic os vesalianum pedis (OVP). (a) Radiograph of the first patient, a 20-year-old male. (b) Radiograph of the second patient, a 43-year-old female. Both images demonstrate a well-corticated ossicle with smooth, rounded margins located proximal to the base of the fifth metatarsal

Initial plain radiographs revealed small bony fragments proximal to the base of the fifth metatarsal, which led to the preliminary diagnosis of an avulsion fracture. However, a detailed analysis by the specialist highlighted features inconsistent with acute trauma. The presence of a well-corticated, bean-shaped ossicle with smooth, rounded margins ultimately led to the correct diagnosis of OVP in both cases (Fig. 1).

At the time of presentation, the *Foot and Ankle Disability Index* (FADI) score was 60% in the first patient and 65% in the second. Both patients were managed conservatively with the rest, ice, elevation, physiotherapy and *Non-Steroidal Anti-Inflammatory Drugs* (NSAIDs) for seven days.

At a three-week follow-up, both patients reported complete symptom resolution. The clinical examination was unremarkable, and the FADI score for both patients had improved to 100%, thus confirming a full functional recovery.

## Discussion

Historically, OVP has been recognized for centuries, first described by Andreas Vesalius in the 16^th^ century, and later formally named by the German anatomist Pfitzner [1]. OVP is a rare anatomical variation of the foot [2–5,9]. Studies by Candan et al. and Coskun et al. independently compared the incidence of accessory bones in the foot, reporting that among the most commonly identified variants are the os trigonum (2.3–9.8%), the accessory navicular (7.9–11.7%), and the os peroneum (4.7–5.8%) [2, 9]. In contrast, OVP has a significantly lower reported incidence of approximately 0.6% [2,9].

The prevailing hypothesis suggests that OVP originates from a secondary ossification center associated with the tuberosity of the fifth metatarsal that does not unite with the main metatarsal shaft during skeletal development [3]. Normally, this apophyseal center becomes visible on radiographs around age 10 in girls and age 12 in boys, with fusion typically occurring within two to four years. When this fusion process is incomplete, the ossicle remains as an independent bony structure into adulthood [3].

We report two cases of OVP both initially misdiagnosed as fifth metatarsal fractures after trauma. This diagnostic confusion is not uncommon. Mathew et al. described a unilateral symptomatic OVP initially mistaken for an avulsion fracture [1]. Similarly, Mousafeiris et al. reported bilateral OVP misdiagnosed as a fracture of the fifth metatarsal [6]. De Castro Correia et al. documented a case of bilateral OVP in a professional footballer with pain on only one side and no previous history of trauma [8]. Dorrestijn and Brouwer presented a complex case involving bilateral painful OVP, which began unilaterally after minor trauma and later progressed to severe contralateral pain without a clear trigger, eventually requiring surgical excision [10]. Furthermore, Petrera et al. described a golf player with slowly progressive lateral right foot pain, with imaging also revealing asymptomatic OVP on the left side. Failure of conservative treatment in this case ultimately led to surgery [11].

OVP is frequently asymptomatic and discovered incidentally on radiographs obtained after trauma [6]. Although rare, symptomatic OVP is an important cause of lateral foot pain [1,6–8,10,11].

Known triggers for symptom onset include trauma, bone degeneration, inflammation, or infection [6,7]. A key diagnostic challenge lies in its radiographic similarity to more common and urgent conditions, such as acute avulsion fractures of the fifth metatarsal [8]. This overlap underscores the importance of clinical vigilance and a thorough understanding of the anatomical and imaging features that distinguish OVP from other entities [1,11].

The central difficulty in diagnosing OVP lies in attributing clinical symptoms to the correct source. In trauma cases with lateral foot pain, incidental identification of OVP on radiographs can complicate diagnostic reasoning [12]. It is often misinterpreted as a fracture at the base of the fifth metatarsal, leading to unnecessary treatments such as immobilization or casting [12]. Conversely, assigning symptoms to a radiographically apparent but asymptomatic OVP may obscure other diagnoses, such as peroneal tendinopathy or stress fractures, thus delaying appropriate treatment and prolonging morbidity [12].

Correctly identifying OVP carries significant clinical implications, as it enables differentiation from a broad range of other causes of lateral foot pain [8]. To assist in the accurate identification of accessory ossicles such as OVP, Kunc et al. [13] proposed specific radiologic criteria, which include: (1) a well-defined, regular oval shape; (2) smooth, corticated margins; and (3) a uniform cortical-to-medullary bone ratio that mirrors normal bone structure which were further expanded by Gaydarski et al. [14], with the addition of no history or signs of prior trauma, especially childhood trauma, and the absence of fibrosis between the fragments. The differential diagnosis is extensive and should consider the age and underlying pathology, including fractures, apophysitis, and other anatomical variants [8]. Conditions commonly included in the differential diagnosis are avulsion (pseudo-Jones) fracture, Jones fracture, stress fracture, Iselin’s disease, normal apophysis, and other accessory ossicles [1,15–17]. These entities are systematically compared in Table 1 with respect to patient age, etiology, clinical features, and imaging findings (Table 1).

**Table 1:** Differential diagnosis of symptomatic OVP

Condition	Typical Age	Etiology	Clinical Presentation	Radiographic Features
**Symptomatic OVP**	Any age after skeletal maturity	Congenital variant, becomes symptomatic after acute trauma or chronic overuse	Localized pain and tenderness at the 5^th^ metatarsal base, exacerbated by resisted eversion	Well-corticated, round/oval ossicle proximal to 5^th^ metatarsal tuberosity. Separated by a smooth, uniform synchondrosis. May be bilateral [1]
**Avulsion (Pseudo-Jones) Fracture**	Adults	Acute inversion injury causing avulsion by peroneus brevis tendon	Acute onset of pain, swelling, and tenderness at the 5^th^ metatarsal tuberosity after injury	Transverse fracture line, sharp, irregular, non-corticated margins. May extend into the tarsometatarsal joint. Unilateral [15]
**Jones Fracture**	Adults	Acute adduction force or chronic stress	Pain localized to the metaphyseal-diaphyseal junction, distal to the tuberosity	Transverse fracture line at the metaphyseal-diaphyseal junction (1.5–2 cm distal to tuberosity). High risk of non-union [16]
**Diaphyseal Stress Fracture**	Adults, athletes	Chronic overuse/repetitive loading	Insidious onset of aching pain in the proximal shaft of the 5^th^ metatarsal, worsens with activity	Often negative initially. Later shows periosteal reaction, cortical thickening, or a faint transverse fracture line distal to a Jones fracture [17]
**Iselin’s Disease (Traction Apophysitis)**	Adolescents (Girls: 8–11, Boys: 11–14)	Overuse injury; repetitive traction on the unfused apophysis by the peroneus brevis	Gradual onset of pain at the 5^th^ metatarsal base in a young athlete. Resolves with skeletal maturity	Apophysis appears widened, irregular, or fragmented. Parallel orientation to the metatarsal shaft. MRI shows edema in the apophysis [1]
**Normal Apophysis**	Adolescents (appears at age 10–12, fuses at age 14–16)	Normal skeletal development	Asymptomatic incidental finding	Small, shell-shaped ossification center oriented parallel to the long axis of the metatarsal. Smooth margins. No associated swelling or edema [15]
**Os Peroneum**	Any age	Sesamoid bone within the peroneus longus tendon	Can be asymptomatic or cause pain (painful os peroneum syndrome) more proximally, near the cuboid	Bean-shaped ossicle located more proximally and plantarly than OVP, adjacent to the cuboid/calcaneocuboid joint. Prominently more common than OVP [1]

Other accessory ossicles should also be considered as potential etiological factors in cases of unexplained foot pain, particularly os naviculare and os trigonum, which are among the most frequently encountered anatomical variants in the foot [2,9]. For example, Slavchev and Georgiev reported a case involving posterolateral ankle pain in a female patient with no history of trauma, ultimately diagnosed as symptomatic os trigonum [18]. Similarly, Georgiev and Stokov described a patient presenting with medial foot pain attributed to an accessory navicular bone [19]. In a broader review, Kotov et al. summarized multiple cases of medial foot pain linked to os naviculare, where conservative treatment had failed [20]. These reports emphasize the importance of including accessory ossicles such as os vesalianum pedis in the differential diagnosis when evaluating foot pain, especially in the absence of clear traumatic injury, so that to avoid misdiagnosis and ensure appropriate management [20].

In any patient presenting with lateral foot pain, OVP should be considered in the differential diagnosis. Differentiation from atypical fractures must be guided by the radiologic criteria established by Gaydarski et al. [14]. Standard radiographic imaging is the first-line diagnostic modality [1,6,8,10,11], and imaging of the contralateral foot is essential due to the frequently bilateral nature of OVP [1,6,8,10,11]. In complex cases, advanced imaging techniques such as computed tomography (CT) and bone scintigraphy may be required [10]. Magnetic resonance imaging (MRI) is particularly useful in assessing soft tissue involvement, including bone marrow edema and peroneal tendon pathology [1].

Initial management of symptomatic OVP should always be conservative [1,6,8,10,11]. Standard conservative measures include rest, ice, compression, elevation, physiotherapy, and NSAIDs [1,6,8]. Functional outcomes and disability levels can be monitored by using the FADI, which is a validated tool for assessing limitations related to foot and ankle disorders [21]. In patients with chronic, refractory symptoms who are unresponsive to conservative treatment, surgical excision of OVP may be indicated [10,11]. This procedure should involve meticulous attention to the repair or reattachment of the peroneus brevis tendon, and it has been shown to be an effective second-line option [10,11].

## Limitations

The present case report has several limitations that should be acknowledged.

- The diagnostic process for both patients relied exclusively and only on plain radiographs without lateral view radiographs. Advanced imaging, such as CT or MRI, was not performed. These modalities could have provided a more detailed assessment of the ossicle’s structure and the condition of surrounding soft tissues like the peroneal tendon.
- Radiographs of the contralateral (unaffected) foot were not obtained for comparison. This is a limitation because OVP is frequently bilateral, and imaging the other foot is considered essential for a comprehensive evaluation. However, we chose not do X-ray of the asymptomatic foot due to the absence of clinical indications. This decision aligns with the *As Low As Reasonably Achievable* (ALARA) principle of minimizing radiation exposure by avoiding medically unnecessary imaging.
- The follow-up period was limited to three weeks. While both patients achieved full functional recovery in this timeframe, a longer-term follow-up would be necessary to assess the potential for symptom recurrence.

## Conclusion

OVP is a rare accessory ossicle that can be easily mistaken for an acute fracture, particularly in the context of lateral foot pain following trauma. Misdiagnosis may lead to unnecessary or inappropriate treatment. Our two cases highlight the importance of recognizing OVP based on its characteristic imaging features, well-corticated, smoothly contoured ossicles near the base of the fifth metatarsal. A thorough clinical examination combined with careful radiological assessment is essential for accurate diagnosis. Conservative treatment, including rest, ice, elevation, physiotherapy, and NSAIDs, proved effective, resulting in full functional recovery within three weeks. Increased awareness of OVP among clinicians is crucial for ensuring proper management and avoiding diagnostic errors.

## References

[1] Mathew AJ, Hugh C, Nath S, Pillai MG (2023). Red Herring in Orthopedics: A Case Report on Painful Os Vesalianum Pedis Masquerading as an Avulsion Fracture of 5th Metatarsal and Review of Literature. *J Orthop Case Rep*.

[2] Candan B, Torun E, Dikici R (2022). The Prevalence of Accessory Ossicles, Sesamoid Bones, and Biphalangism of the Foot and Ankle: A Radiographic Study. *Foot Ankle Orthop*.

[3] Birrer RB, Griesemer B, Cataletto ME (2002). Pediatric sports medicine for Primary Care.

[4] Osiowski A, Preinl M, Osiowski M, Baran K, Jasiewicz B, Taterra D (2025). The prevalence and clinical considerations of Os Vesalianum Pedis: A meta-analysis. *Foot Ankle Surg*.

[5] Keles-Celik N, Kose O, Sekerci R, Aytac G, Turan A, Güler F (2017). Accessory Ossicles of the Foot and Ankle: Disorders and a Review of the Literature. *Cureus*.

[6] Mousafeiris VK, Papaioannou I, Kalyva N, Arachoviti C, Repantis T (2021). Os Vesalianum Pedis in a Young Adult: A Case Report and Literature Review. *Cureus*.

[7] Wilson TC, Wilson RC, Ouzounov KG (2011). The symptomatic os vesalianum as an uncommon cause of lateral foot pain: a case report. *J Am Podiatr Med Assoc*.

[8] De Castro Correia M, Rodrigues Lopes T (2022). Knowing Your Accessory Foot Ossicles and Avoiding Misdiagnoses: A Case Report of Painful Os Vesalianum Pedis. *Cureus*.

[9] Coskun N, Yuksel M, Cevener M (2009). Incidence of accessory ossicles and sesamoid bones in the feet: a radiographic study of the Turkish subjects. *Surg Radiol Anat*.

[10] Dorrestijn O, Brouwer RW (2011). Bilateral symptomatic os vesalianum pedis: a case report. *J Foot Ankle Surg*.

[11] Petrera M, Dwyer T, Ogilvie-Harris DJ (2013). A rare cause of foot pain with golf swing: symptomatic os vesalianum pedis-a case report. *Sports Health*.

[12] Kose O (2009). Os vesalianum pedis misdiagnosed as fifth metatarsal avulsion fracture. *Emerg Med Australas*.

[13] Kunc V, Kunc V, Černý V, Polovinčák M, Kachlík D (2020). Accessory bones of the elbow: Prevalence, localization and modified classification. *J Anat*.

[14] Gaydarski L, Mirazchiyski G, Panev A The elusive bipartite scaphoid: a rare congenital variant or misdiagnosed pseudoarthrosis? A proposal for novel radiological criteria. *Folia Morphol (Warsz)*.

[15] Vaz A, Trippia CR (2018). Small but troublesome: accessory ossicles with clinical significance. *Radiol Bras*.

[16] Uğuz Gençer C, Tetiker H, Koşar MI, Çullu N (2024). The Prevalence and the Clinical Importance of os vesalianum pedis. Prevalence a klinický význam os vesalianum pedis. *Acta Chir Orthop Traumatol Cech*.

[17] Cruz MF, Bolgla LA, Alexander R, Symbas PJ, Royal J, Serrano-Dennis J (2023). Conservative treatment of a symptomatic os vesalianum pedis in a professional soccer player: a case report. *Internet J Allied Health Sci Pract*.

[18] Slavchev SA, Georgiev GP (2014). Os trigonum: a case report of a symptomatic anatomical variation and its surgical treatment. *Rev Argent Anat Clin*.

[19] Georgiev GP, Stokov L (2010). Surgical treatment of the accessory navicular bone: case report. *J Biomed Clin Res*.

[20] Kotov G, Iliev A, Landzhov B, Dimitrova IN, Slavchev S, Georgiev GP (2016). A clinical, radiographic and histological study of the accessory navicular bone. *Praemedicus*.

[21] Martin RL, Burdett RG, Irrgang JJ (1999). Development of the Foot and Ankle Disability Index (FADI) [abstract]. *J Orthop Sports Phys Ther*.

